# Psychiatric-disorder-related behavioral phenotypes and cortical hyperactivity in a mouse model of 3q29 deletion syndrome

**DOI:** 10.1038/s41386-019-0441-5

**Published:** 2019-06-19

**Authors:** Masayuki Baba, Kazumasa Yokoyama, Kaoru Seiriki, Yuichiro Naka, Kensuke Matsumura, Momoka Kondo, Kana Yamamoto, Misuzu Hayashida, Atsushi Kasai, Yukio Ago, Kazuki Nagayasu, Atsuko Hayata-Takano, Akinori Takahashi, Shun Yamaguchi, Daisuke Mori, Norio Ozaki, Tadashi Yamamoto, Kazuhiro Takuma, Ryota Hashimoto, Hitoshi Hashimoto, Takanobu Nakazawa

**Affiliations:** 1 Laboratory of Molecular Neuropharmacology, Graduate School of Pharmaceutical Sciences, Osaka University, Suita, Osaka 565-0871 Japan; 2 Pharmaceutical Research Division, Takeda Pharmaceutical Company Limited, Kanagawa Fujisawa, 251-8555 Japan; 3 Interdisciplinary Program for Biomedical Sciences, Institute for Transdisciplinary Graduate Degree Programs, Osaka University, Suita, Osaka 565-0871 Japan; 4 Research Fellowships for Young Scientists of the Japan Society for the Promotion of Science, Chiyoda-ku, Tokyo, 102-0083 Japan; 5 Laboratory of Biopharmaceutics, Graduate School of Pharmaceutical Sciences, Osaka University, Suita, Osaka 565-0871 Japan; 6 Molecular Research Center for Children’s Mental Development, United Graduate School of Child Development, Osaka University, Suita, Osaka 565-0871 Japan; 7 Cell Signal Unit, Okinawa Institute of Science and Technology Graduate University, Onna-son, Okinawa, 904-0495 Japan; 8 Department of Morphological Neuroscience, Gifu University Graduate School of Medicine, Gifu, 501-1194 Japan; 9 Center for Highly Advanced Integration of Nano and Life Sciences, Gifu University, Gifu, 501-1194 Japan; 10 Department of Psychiatry, Nagoya University Graduate School of Medicine, Aichi, Nagoya, 466-8550 Japan; 11 Brain and Mind Research Center, Nagoya University, Aichi, Nagoya, 466-8550 Japan; 12 Laboratory for Immunogenetics, Center for Integrative Medical Sciences, RIKEN, Kanagawa Yokohama, 230-0045 Japan; 13 Department of Pharmacology, Graduate School of Dentistry, Osaka University, Suita, Osaka 565-0871 Japan; 14 Department of Pathology of Mental Diseases, National Institute of Mental Health, National Center of Neurology and Psychiatry, Kodaira, Tokyo, 187-8553 Japan; 15 Osaka University, Suita, Osaka 565-0871 Japan; 16 Division of Bioscience, Institute for Datability Science, Osaka University, Suita, Osaka 565-0871 Japan; 17 Transdimensional Life Imaging Division, Institute for Open and Transdisciplinary Research Initiatives, Osaka University, Suita, Osaka 565-0871 Japan

**Keywords:** Schizophrenia, Schizophrenia, Autism spectrum disorders

## Abstract

3q29 microdeletion, a rare recurrent copy number variant (CNV), greatly confers an increased risk of psychiatric disorders, such as schizophrenia and autism spectrum disorder (ASD), as well as intellectual disability. However, disease-relevant cellular phenotypes of 3q29 deletion syndrome remain to be identified. To reveal the molecular and cellular etiology of 3q29 deletion syndrome, we generated a mouse model of human 3q29 deletion syndrome by chromosome engineering, which achieved construct validity. 3q29 deletion (Df/+) mice showed reduced body weight and brain volume and, more importantly, impaired social interaction and prepulse inhibition. Importantly, the schizophrenia-related impaired prepulse inhibition was reversed by administration of antipsychotics. These findings are reminiscent of the growth defects and neuropsychiatric behavioral phenotypes in patients with 3q29 deletion syndrome and exemplify that the mouse model achieves some part of face validity and predictive validity. Unbiased whole-brain imaging revealed that neuronal hyperactivation after a behavioral task was strikingly exaggerated in a restricted region of the cortex of Df/+ mice. We further elucidated the cellular phenotypes of neuronal hyperactivation and the reduction of parvalbumin expression in the cortex of Df/+ mice. Thus, the 3q29 mouse model provides invaluable insight into the disease-causative molecular and cellular pathology of psychiatric disorders.

## Introduction

Accumulating evidence suggests that psychiatric disorders are strongly associated with rare variants with high penetrance, in addition to the potential cumulative effect of a large number of common genetic variants with small individual effects [1–3]. The most well-characterized forms of rare but recurrent variations are copy number variants (CNVs), which represent large genomic duplications or deletions. Rare but recurrent CNVs have been widely considered to contribute to genetic vulnerability to psychiatric disorders, including schizophrenia and autism spectrum disorders (ASDs) [4–7]. A recent genome-wide study has shown that eight loci, including 1q21.1 (deletion and duplication), 2p16.3 (NRXN1) (deletion), 3q29 (deletion), 7q11.23 (duplication), 15q13.3 (deletion), distal 16p11.2 (deletion), proximal 16p11.2 (duplication), and 22q11.2 (deletion), are associated with a high risk for psychiatric disorders (odds ratio, 3.8-infinite) [8]. Importantly, approximately 2.5% of patients with schizophrenia carry at least one of these CNVs [9].

The 3q29 microdeletion is mostly de novo and the incidence for the microdeletion is 1 in 30,000–40,000 birth [5, 10–13]. The 3q29 microdeletion is typically approximately 1.6 Mb in size and contains approximately 22 genes. The 3q29 microdeletion is associated with ASD, bipolar disorder, intellectual disability, and schizophrenia [13–17]. Particularly, among the schizophrenia-associated CNVs, the 3q29 microdeletion is likely to confer the greatest risk for schizophrenia (40 to infinite increase in risk) [13]. In addition to these diseases, the 3q29 microdeletion is also associated with speech delay, anxiety, and microcephaly [5]. To date, while a mouse model of 3q29 deletion syndrome has been reported very recently [18], there are few studies on the effect of the 3q29 microdeletion on neural function. Accordingly, disease-relevant cellular phenotypes of 3q29 deletion syndrome are almost completely unclear.

Psychiatric disorders, such as schizophrenia and ASD, share, in part, genetic causes, pathophysiology, and clinical symptoms. Central nervous development and neuronal circuit functions are commonly impaired in patients with these disorders. In particular, multiple lines of evidence suggest that an altered cellular balance of excitation and inhibition (E/I balance) within neural circuitry is a shared pathophysiological mechanism for schizophrenia and ASD [19]. Consistent with this, studies of postmortem brains show that GABAergic interneuron density and distribution are altered in patients with schizophrenia [20–24]. Importantly, the expression of GABA-related molecules, including the GABA-synthesizing enzymes GAD67, GABA membrane transporter GAT1, GABA receptor subunits, parvalbumin (PV), somatostatin, cholecystokinin, neuropeptide Y, calretinin, and calbindin, is altered in patients with schizophrenia [22, 25, 26]. In addition to schizophrenia, GABAergic neurotransmission is suggested to be impaired in patients with ASD [27]. Because GABAergic interneurons regulate a number of brain functions that govern cognitive and emotional behaviors [22, 28, 29], impaired brain functions in patients with psychiatric disorders is in part caused by abnormalities in the GABAergic system.

Given that rare recurrent pathological CNVs of patients with psychiatric disorders show extraordinarily high penetrance [8], genetically engineered mice harboring these CNVs are important models for investigation of relevant phenotypes associated with psychiatric disorders. In this paper, to study the impact of the 3q29 microdeletion on neuronal functions in vivo, we generated genetically engineered mice harboring a deletion of the chromosomal region corresponding to the human 3q29 region through Cre-mediated recombination. *Del(16Bdh1-Tfrc)/*+ mice (heterozygous 3q29 deletion mice; hereafter Df/+ mice) showed slightly reduced body weight as well as brain volume. Df/+ mice also showed psychiatric disorder-related behavioral phenotypes in the schizophrenia-related prepulse inhibition (PPI) test, ASD-related reciprocal interaction test and self-grooming test, and contextual fear conditioning test. Importantly, impaired PPI was reversed by risperidone treatment. Using FAST (block-FAce Serial microscopy Tomography), a high-speed serial-sectioning imaging system that allows us to unbiasedly analyze whole-brain neural activity [30, 31], we found that neuronal activity was abnormally activated in a restricted region of the cortex of Df/+ mice. In the cortex of Df/+ mice, we found that the expression levels of immediate early genes were increased and that the number of PV-positive neurons was decreased. Taken together, our current findings suggest that heterozygous 3q29 deletion impairs the GABAergic system, leading to increased cortical neuronal activity and psychiatric disorder-related behavioral phenotypes. Df/+ mice can help better understand the disease-causative molecular etiology of psychiatric disorders and develop new and better treatments for psychiatric disorders.

## Materials and methods

All procedures were performed in accordance with the standards of humane care, and the treatment of the research animals was approved by the Institutional Animal Care and Use Committee in Takeda Pharmaceutical Company Limited, Osaka University and Okinawa Institute of Science and Technology Graduate University. All recombinant DNA experiments were reviewed and approved by the Gene Modification Experiments Safety Committee of Takeda Pharmaceutical Company Limited and Osaka University.

### Generation of a mouse model of 3q29 syndrome

The common breakpoint regions in human 3q29 deletion syndrome were analyzed with NCBI BLAST to find region-specific low-copy repeats (LCRs), which may facilitate 3q29 microdeletion [11]. Genomic coordinates of the LCRs in UCSC hg19 were converted to mouse genomic coordinates in UCSC mm10 with liftOver. The human 3q29 region between LCRs corresponds to mouse chromosome 16: 31,336,396–32,632,800. We constructed two targeting vectors and two pairs of CRISPR single guide RNAs to insert loxPs into mouse chromosome 16: 31,415,600, just proximal to *Bdh1*, and 32,632,500, just distal to *Tfrc*, with the hygromycin resistance gene and the puromycin resistance gene, respectively. These vectors, along with a Cas9 nickase plasmid, were electroporated into embryonic stem (ES) cells derived from C57BL/6J mice and selected successively with hygromycin and puromycin. We utilized the Cas9 nickase (D10A) mutant to reduce off-target mutagenesis [32, 33]. Selected clones were transiently transfected with a Cre recombinase plasmid and tested for copy number analysis of the *Bdh1*-*Tfrc* region by genome quantitative PCR (qPCR) analysis, by which ES cells harboring two loxP sites in *cis* resulted in a chromosome deletion and apparent reduction in the genome copy number of the *Bdh1*-*Tfrc* region, whereas cells harboring two loxP sites in *trans* resulted in a balanced duplication and deletion and unaltered genome copy number. In this study, a deleted ES clone was injected into tetraploid blastocysts of the ICR strain, and chimeric mice were bred with C57BL/6J female mice to generate *Del(16Bdh1-Tfrc)/*+ mice (heterozygous 3q29 deletion mice; Df/+ mice). Transmission of a *Bdh1*-*Tfrc* deletion was confirmed by genome qPCR.

### RNA sequence

Total RNA isolated from 8-week-old mouse brains was sequenced using an Illumina system (Macrogen Inc., Seoul, Korea). UCSC mm10 was used as a reference genome to map complementary DNA fragments obtained from RNA-sequencing. The fragments per kilobase of transcript per million mapped reads value was used as the expression profile. The significant results were selected on conditions of |FC| >2 and independent *t* test raw *P* < 0.05. Based on these criteria, seven genes were differentially expressed between Df/+ mice and wild-type (WT) littermates (*n* = 3).

### Behavioral analysis

All behavioral analyses were carried out on male C57BL/6J mice at approximately 2 months of age. Three different mouse batches were used for behavioral analyses. The first one was used for the self-grooming test, social interaction test, and PPI test in this order. The second one was used for the open-field test and contextual fear conditioning test in this order. The last one was used for the PPI test with risperidone. All behavioral analyses were performed during the light period in a completely blinded manner.

### PPI test

A startle chamber (SR-LAB, San Diego Instruments, CA, USA) was used to assess the acoustic startle responses and PPI as described previously [34]. Briefly, after a background noise of 65 dB was presented for 5 min, the following experimental sessions were performed. Each mouse was exposed to four consecutive blocks with a total of 100 trials within approximately 30 min. One block consisted of five different trial types, including pulse-alone trials (40 ms broadband 120 dB burst), three different prepulse-pulse trials (3, 6, and 9 dB prepulse intensities above the 65 dB background noise and 120 dB startle pulse) and no-stimulation trials. The startle response was recorded for 100 ms. The percentage of PPI was calculated as [(startle amplitude without prepulse) – (startle amplitude with prepulse)]/(startle amplitude without prepulse) × 100. With this standard method, impaired PPI is suggested to be relevant to schizophrenia, but not to ASD [7].

### Reciprocal social interaction test

The reciprocal social interaction test was performed as described previously [35–37]. Briefly, a male intruder mouse was placed in the test cage after habituation of the test mouse to the same cage for 60 min. Over the whole experimental period (20 min), the total duration of time that the resident mouse spent sniffing, following, allo-grooming, and push-crawling the intruder was measured.

### Self-grooming test

After a 10-min period of habituation to a test cage, the cumulative time that the test mouse spent grooming itself in the test cage was manually measured for 10 min.

### Contextual fear conditioning test

Fear conditioning was conducted in a fear conditioning chamber (30 × 24 × 21 cm^3^, Med Associates Inc., VT, USA). Each mouse was placed in the chamber for 120 s, and then the mouse was given a footshock (5 s/ 0.8 mA) (4 times with a 30-s intershock interval). On the test day, the mouse was placed in the same chamber as used in the conditioning. Then, the freezing response was measured for 6 min. Freezing was monitored by Med Associates Video Freeze software.

### Open-field test

Locomotor activity was measured as described previously [38]. Briefly, each mouse was placed in the center of the apparatus (45 × 45 × 30 cm^3^). The total distance traveled and time spent in the center zone (25 × 25 cm^2^) were recorded for 90 and 5 min, respectively, per mouse.

### Real-time PCR

Total RNA isolated from cortices was reverse transcribed with Superscript III (Life Technologies, CA, USA). Using SYBR Premix EX Taq (Takara Bio Inc., Shiga, Japan), real-time PCR was performed with a CFX96 real-time PCR detection system (Bio-Rad Laboratories, CA, USA) according to the manufacturer’s instructions. The expression levels of each gene were normalized to those of *Gapdh* and were determined according to the 2^-∆∆Ct^ method. The primers used were as follows: *Egr2*, 5′-CTACCCGGTGGAAGACCTC-3′ and 5′-AATGTTGATCATGCCATCTCC-3′; *Fos*, 5′-GGCTCTCCTGTCAACACACA-3′ and 5′-GACCAGAGTGGGCTGCAC-3′; *Cyr61*, 5′-GCCAAGAAGAATAAGAGATGTGATTA-3′ and 5′-AGGGGGCTTGGAGTCCTT-3′; *Nr4a1*, 5′-GGGGGAGTGTGCTAGAAGG-3’ and 5′-TTGAATACAGGGCATCTCCA-3′; *Btg2*, 5′-GTTTTCAGTAGGGCGCTCCAGGAC-3′ and 5′-TGGTTGATACGGATACAGCGATAG-3′; *Dusp1*, 5′-CTGGAGAACCGCAGAACG-3′ and 5′-GATGCCCACCTCCATCAC-3′; *Gapdh*, 5′-GTGTTCCTACCCCCAATGTG-3′ and 5′-TACCAGGAAATGAGCTTGAC-3′.

### Administration of risperidone

Risperidone (Sigma-Aldrich, MO, USA) dissolved in saline (0.9% NaCl solution) containing <0.1% (v/v) acetic acid was intraperitoneally administered to adult male mice at a dosage of 1 mg/kg 30 min before the PPI test.

### FAST whole-brain imaging

Adult male Df/+ mice and WT littermates, both of which were crossed with Arc-dVenus mice [39], were subjected to the reciprocal social interaction test. Five hours after the test, which is the peak time of dVenus expression driven by the Arc promoter, the mice were perfused with 4% paraformaldehyde (PFA) in phosphate-buffered saline (PBS) to fix the brain. Serial whole-brain imaging was performed using FAST, which renders brain-wide anatomical and functional analyses at the single-cell level, including cell-type-specific activity mapping of disease model brains with various reporter animals [30, 31]. Briefly, the mouse brain was embedded in 4% agarose gel (Nacalai tesque, Kyoto, Japan) dissolved in PBS and subjected to automated serial section imaging. Whole-brain images were obtained at a resolution of 1.0 × 1.0 × 5 µm^3^ using FAST with a ×16 NA 0.8 objective lens (Nikon instruments, Tokyo, Japan), a ×0.83 intermediate magnification lens (Yokogawa Electronic, Tokyo, Japan), and an sCMOS camera (Andor Technology, Belfast, UK) with a 2 × 2 binning mode. Quantification of dVenus-positive neurons and semiautomatic anatomical parcellation of the brain were performed using TRI/FCS-NUC64 software (Ratoc System Engineering, Tokyo, Japan).

### Histological analysis

Adult mouse brains were fixed with 4% PFA in PBS overnight at 4°C and were sectioned at a 50 μm thickness using a linear slicer (Linear Slicer Pro7, Dosaka EM, Kyoto, Japan) for PV staining and at a 20 μm thickness using a cryostat (Leica, Wetzlar, Germany, CM1520) for SATB2 staining. For PV staining, the brain slices were permeabilized with blocking solution containing 0.1% Triton X-100 (Wako, Osaka, Japan) and 5% normal goat serum (Thermo Fisher Scientific, Waltham, MA, USA) Tris-buffered saline for 1 h at room temperature and then incubated with the blocking solution combined with primary antibodies. For SATB2 staining, the antigen was retrieved by microwave treatment for 20 min in boiling 10 mM citric acid, pH 7.0, followed by 20 min treatment in Histo VT one (Nacalai Tesque, Kyoto, Japan) at 70 °C. Then, the brain slices were permeabilized with blocking solution containing 0.25% Triton X-100 (Wako, Osaka, Japan), 1% normal goat serum (Thermo Fisher Scientific), and 1% bovine serum albumin (Sigma-Aldrich) in PBS for 1 h at room temperature and then incubated with the blocking solution combined with primary antibodies. The following day, the slices were incubated with the blocking solution combined with secondary antibody for 1 h at room temperature. Images of the slices were acquired using an Olympus FluoView FV1000 confocal microscope (Olympus, Tokyo, Japan) and a BZ-9000 microscope (Keyence, Osaka, Japan). Then, the images were analyzed with ImageJ software (NIH, Bethesda, MD, USA) and Adobe Photoshop CS (Adobe Systems, San Jose, CA, USA). The primary antibodies used were mouse anti-SATB2 (Abcam, Cambridge, UK) and mouse anti-PV (Swant, Marly, Switzerland). The secondary antibodies used were biotinylated goat anti-mouse IgG antibody (Vector Laboratories, Burlingame, CA, USA) and Texas Red streptavidin (Vector Laboratories).

### Statistical analysis

The body weights of Df/+ mice and WT littermates were statistically analyzed using Student’s *t* test and two-way analysis of variance (ANOVA) with repeated measures followed by Bonferroni post hoc tests. The behavioral data were statistically analyzed using one-way ANOVA and two-way ANOVA followed by Bonferroni post hoc tests. The quantified data from the immunohistochemistry were statistically analyzed using Student’s *t* test. The RNA sequence data were statistically analyzed by an unpaired *t* test. The whole-brain imaging data were statistically analyzed by Student’s *t* test. The Benjamin–Hochberg procedure was used to correct *P* value for multiple comparisons. For details, see the description in each figure legend. The significance level was set at *P* < 0.05. Statistical analyses were conducted using Stat-View (SAS Institute, Cary, NC, USA).

## Results

### Generation of a mouse model of 3q29 deletion syndrome

**Fig. 1:**
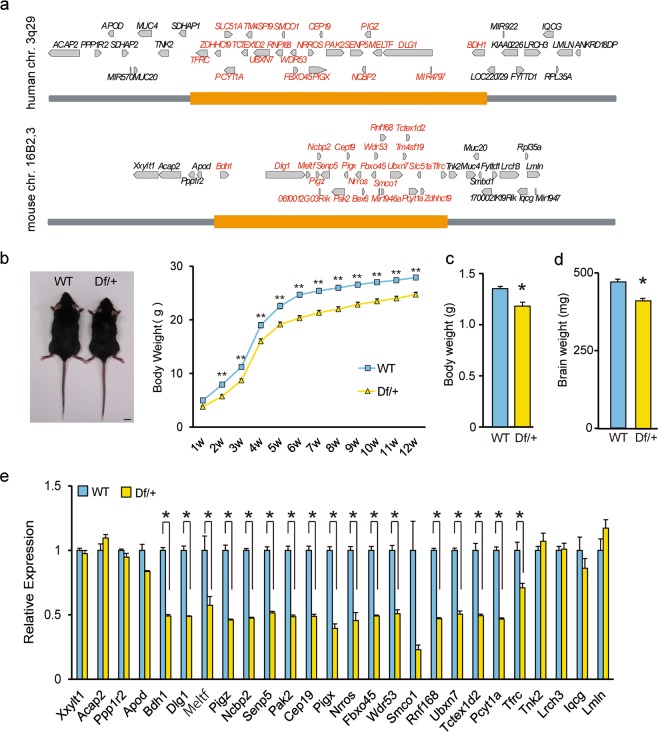
Characterization of Df/+ mice, a mouse model for the human 3q29 deletion. **a** The deleted region in human chromosome 3q29 (top) and the syntenic region in mouse 16B2,3 (bottom). **b** Decreased body size in Df/+ mice (12 weeks old). Scale bar, 1 cm (left). Quantification of body weight in WT and Df/+ mice (WT, *n* = 23; Df/+, *n* = 22) (right). **P < 0.01, repeated two-way analysis of variance (ANOVA). WT, wild-type littermates. **c** Decreased body weight in Df/+ mice (embryonic day 18.5) (WT, *n* = 18; Df/+, *n* = 11). *P < 0.05, Student’s *t* test. **d** Reduced brain weight in Df/+ mice (12 weeks old) (WT, *n* = 10; Df/+, *n* = 10). ***P < 0.001, Student’s *t* test. **e** Messenger RNA (mRNA) expression changes in the cerebral cortex measured by RNA sequencing in 12-week-old Df/+ mice compared to WT mice (each *n* = 3). *Corrected *P* < 0.05, unpaired *t* test with Benjamin–Hochberg false discovery rate correction. Data are presented as the mean ± s.e.m.

Mouse chromosome 16 contains a syntenic region to human chromosome 3q29 with the same gene order (Fig. 1a). To generate a mouse model of 3q29 deletion syndrome, we constructed two targeting vectors to insert loxP sites to the regions in mouse chromosome 16 corresponding to common breakpoints in human 3q29 deletion syndrome [11]: one was proximal to *Bdh1* and the other distal to *Tfrc* (Supplementary Fig. 1). ES cells derived from C57BL/6J mice were introduced with the targeting vectors and chromosome engineered by expressing Cre recombinase. Several clones were tested for copy number analysis of the *Bdh1*-*Tfrc* region by genome qPCR analysis (data not shown). The ES cells harboring the deletion were injected into tetraploid blastocysts of the ICR strain, and chimeric mice were bred with C57BL/6J female mice, resulting in the generation of *Del(16Bdh1-Tfrc)/*+ mice (heterozygous 3q29 deletion mice; hereafter Df/+ mice). To avoid any maternal effects of the deletion, we mated male Df/+ mice with female WT C57BL/6J mice to obtain Df/+ mice and their WT littermates for all experiments except the whole-brain imaging.

Df/+ mice were born at the expected Mendelian ratios (birth ratios from Df/+ and WT mice crosses: WT, 378/678 (55.75 %); Df/+, 300/678 (44.25 %)). To validate the 3q29 deletion mouse model, we analyzed the copy number of genes between the *Bdh1* and *Tfrc* genes by genome qPCR analysis and found that each gene copy number was decreased by approximately 50% in Df/+ mice, as expected (Supplementary Fig. 1b). Df/+ mice survived into adulthood and appeared healthy but were slightly smaller than WT littermates in both sexes (Fig. 1b and data not shown). The significantly reduced body weight of Df/+ mice was evident at embryonic day 18.5 (Fig. 1c). Importantly, Df/+ mice also showed a significant reduction in brain weight (Fig. 1d), all of which is reminiscent of the growth defects and small head size in patients with 3q29 microdeletion syndrome [15]. We then determined the mRNA expression levels of the genes between the *Bdh1* and *Tfrc* genes by quantitative reverse transcription PCR (RT-PCR). The mRNA levels of these genes were decreased by approximately 50% in Df/+ mice compared to those of WT littermates (Fig. 1e). Taken together, our results show that Df/+ mice recapitulate a genomic disorder caused by a rare CNV.

### Psychiatric disease-related behavioral phenotypes in heterozygous 3q29 deletion mice

**Fig. 2:**
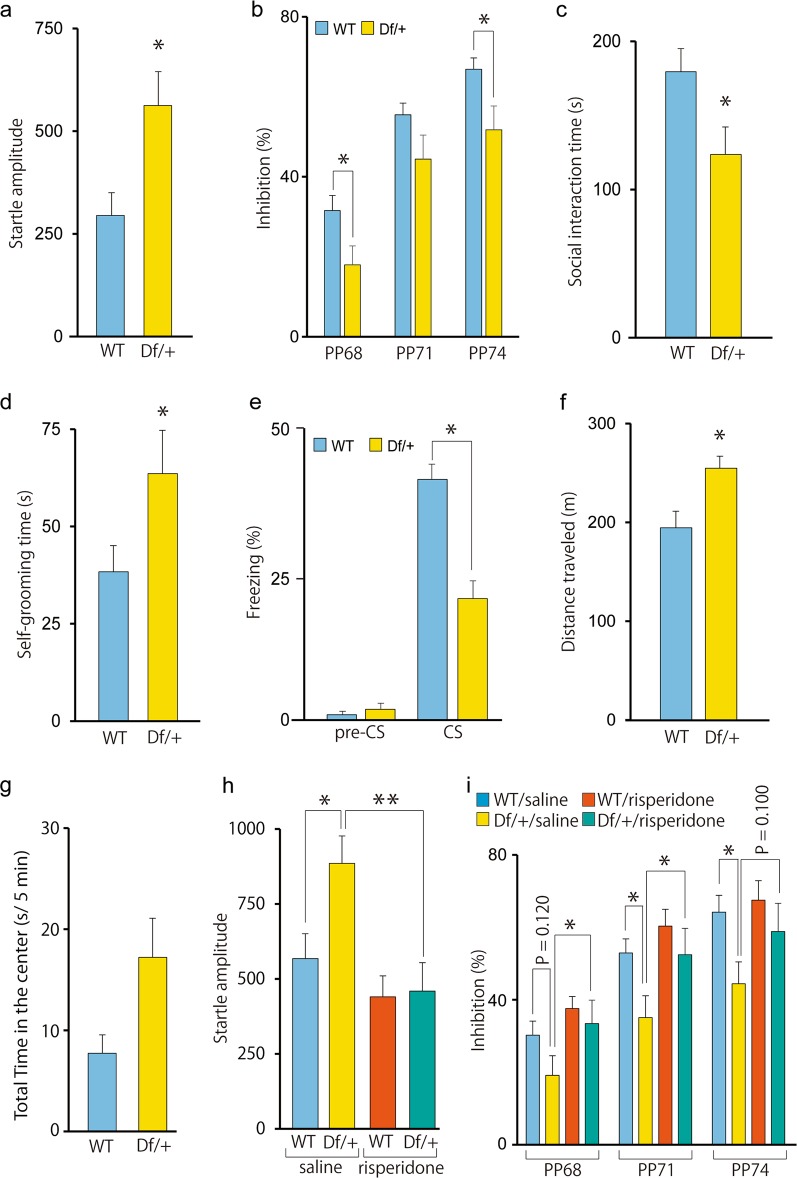
Psychiatric disorder-related behavioral phenotypes in Df/+ mice. **a** Increased startle amplitude in Df/+ mice (WT, *n* = 10; Df/+, *n* = 10). *P < 0.05, one-way analysis of variance (ANOVA). WT, wild-type littermates. **b** Impaired prepulse inhibition (PPI) in Df/+ mice (WT, *n* = 10; Df/+, *n* = 10). *P < 0.05, repeated two-way ANOVA with Bonferroni post hoc tests. **c** Impaired social interaction in Df/+ mice (WT, *n* = 10; Df/+, *n* = 10). *P < 0.05, one-way ANOVA. **d** Increased self-grooming activity (an index of repetitive behavior) in Df/+ mice (WT, *n* = 10; Df/+, *n* = 10). *P < 0.05, one-way ANOVA. **e** Impaired fear conditioning in Df/+ mice (WT, *n* = 10; Df/+, *n* = 9). *P < 0.05, one-way ANOVA. **f** Increased open-field activity in Df/+ mice (WT, *n* = 8; Df/+, *n* = 9). *P < 0.05, one-way ANOVA. **g** Time in the center zone during the first 5 min in the open-field test (WT, *n* = 8; Df/+, *n* = 9). **h** Rescue of the increased startle amplitude by risperidone administration in Df/+ mice (each *n* = 10). *P < 0.05, **P < 0.01, two-way ANOVA with Bonferroni post hoc tests. **i** Rescue of the impaired PPI by risperidone administration in Df/+ mice (each *n* = 10). *P < 0.05, two-way ANOVA with Bonferroni post hoc tests. The percent of the PPI at all dB levels was not significantly different between risperidone-treated Df/+ and WT mice (68 dB, *P* = 0.557; 71 dB, *P* = 0.326; 74 dB, *P* = 0.318). Data are presented as the mean ± s.e.m.

Since 3q29 microdeletion syndrome is associated with ASD and schizophrenia, we performed behavioral tests related to psychiatric disorders. The PPI test provides an operational measure of the sensorimotor gating system, which is impaired in patients with schizophrenia [40]. Before the tests, we measured the startle amplitude of Df/+ mice and WT littermates and determined that the startle response of Df/+ mice was significantly greater than that of WT littermates (Fig. 2a). In Df/+ mice, the percent of the PPI of the startle reflex was significantly lower than that of WT littermates (Fig. 2b), indicating impaired sensory-motor gating. We next performed the reciprocal social interaction and self-grooming tests that model the clinical features of ASD [41]. In the reciprocal social interaction test, in which dyadic pairs of mice freely move and mutually interact with each other in an open arena, Df/+ mice spent less time on social activities, such as sniffing, following, and crawling over/under the partner’s body (Fig. 2c). We then assessed repetitive self-grooming behavior and found that Df/+ mice groomed much more than WT littermates (Fig. 2d). Furthermore, Df/+ mice showed significantly weaker freezing responses in the contextual fear conditioning test, suggesting impaired fear-related learning (Fig. 2e). Additionally, we assessed the activity of Df/+ mice in a novel open field. We found that Df/+ mice showed higher locomotor activity in the novel open field and that time spent in the center zone of Df/+ mice was tended to be longer as compared to that of WT mice; however, the differences did not reach statistical significance (*P* = 0.055) (Fig. 2f, g). Finally, we examined the effect of an atypical antipsychotic, risperidone, on the greater startle response in Df/+ mice and found that the intraperitoneal administration of risperidone effectively rescued the greater startle response in Df/+ mice (Fig. 2h). Likewise, the intraperitoneal administration of risperidone effectively rescued impaired PPI at 68 and 71 dB (*P* = 0.048 and 0.035, respectively), although the effect at 74 dB did not reach statistical significance (*P* = 0.100) (Fig. 2i). Additionally, the percent of the PPI at 71 and 74 dB was significantly lower in saline-treated Df/+ mice as compared to that in saline-treated WT mice (*P* = 0.031 and 0.027, respectively), although the percent of the PPI at 68 dB was not significantly different between saline-treated Df/+ and WT mice (*P* = 0.120). The percent of the PPI at all dB levels was not significantly different between risperidone-treated Df/+ and WT mice (68 dB, *P* = 0.557; 71 dB, *P* = 0.326; 74 dB, *P* = 0.318). Regarding apparent difference of statistical significance of the PPI test between samples in Fig. 2b and saline-injected samples in Fig. 2i, injection stress might be attributable to the difference, although the precise reason for the discrepancy is currently unknown.

### Abnormally increased excitatory neural activity in the cerebral cortex of Df/+ mice

**Fig. 3:**
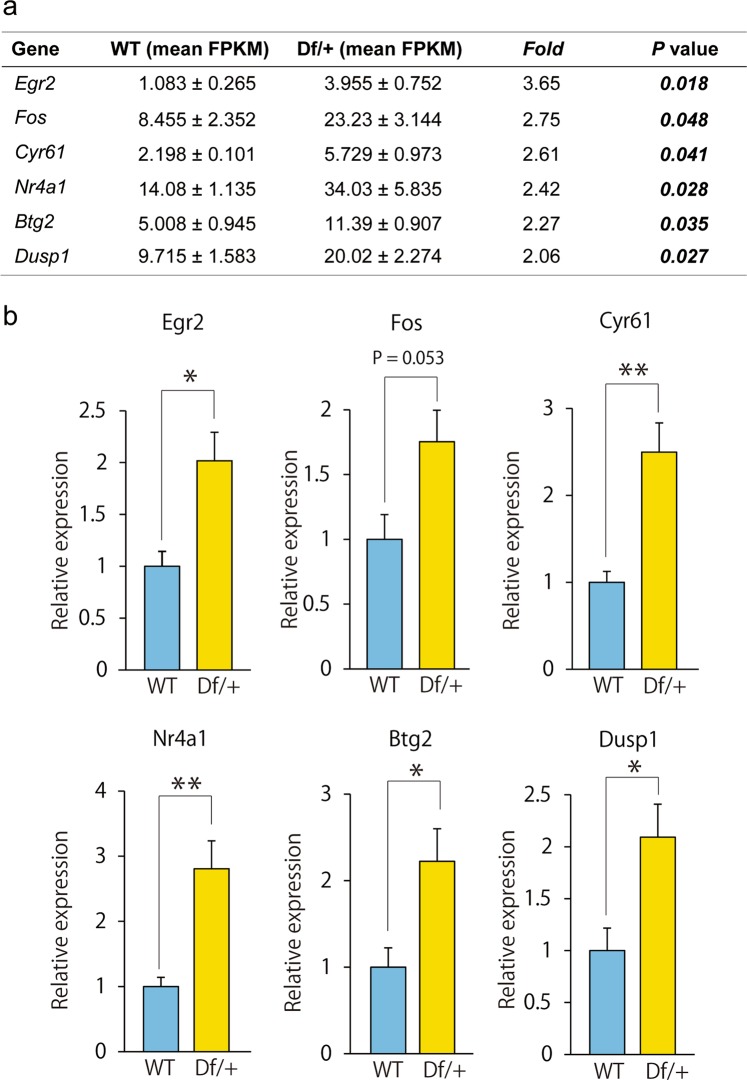
Increased expression of immediate early genes in the cortex of Df/+ mice. **a** Top-ranked differentially expressed genes in the mouse cortex between wild-type (WT) and Df/+ mice (RNA sequence, |FC| > 2, *P* < 0.05) (WT, *n* = 3; Df/+, *n* = 3). Note that the deleted genes in the 16B2, 3 region were not listed in the table (see Fig. 1e). **b** The expression of each immediately early gene was assayed by reverse transcription PCR (RT-PCR) using total RNA from the cerebral cortex (WT, *n* = 4; Df/+, *n* = 5). *P < 0.05, **P < 0.01, Student’s *t* test. Data are presented as the mean ± s.e.m.

Dysfunction of the cerebral cortex is critically involved in impaired social interaction and PPI in mice [42, 43]. Using total RNA obtained from the cerebral cortex of Df/+ mice and WT littermates, we next conducted RNA sequence analysis to explore the molecular mechanisms behind the behavioral phenotypes. Interestingly, top-ranked differentially expressed genes in the cortex between adult Df/+ mice and WT littermates (|FC| > 2, *P* < 0.05), namely, *Egr2*, *Fos*, *Cyr61*, *Nr4a1*, *Btg2*, and *Dusp1* were immediate early genes, all of which showed significantly increased expression in Df/+ mice (Fig. 3a). The increased expression of these immediate early genes was confirmed by RT-PCR analysis (Fig. 3b), further supporting that the excitatory neural activity is abnormally increased in Df/+ mice.

**Fig. 4:**
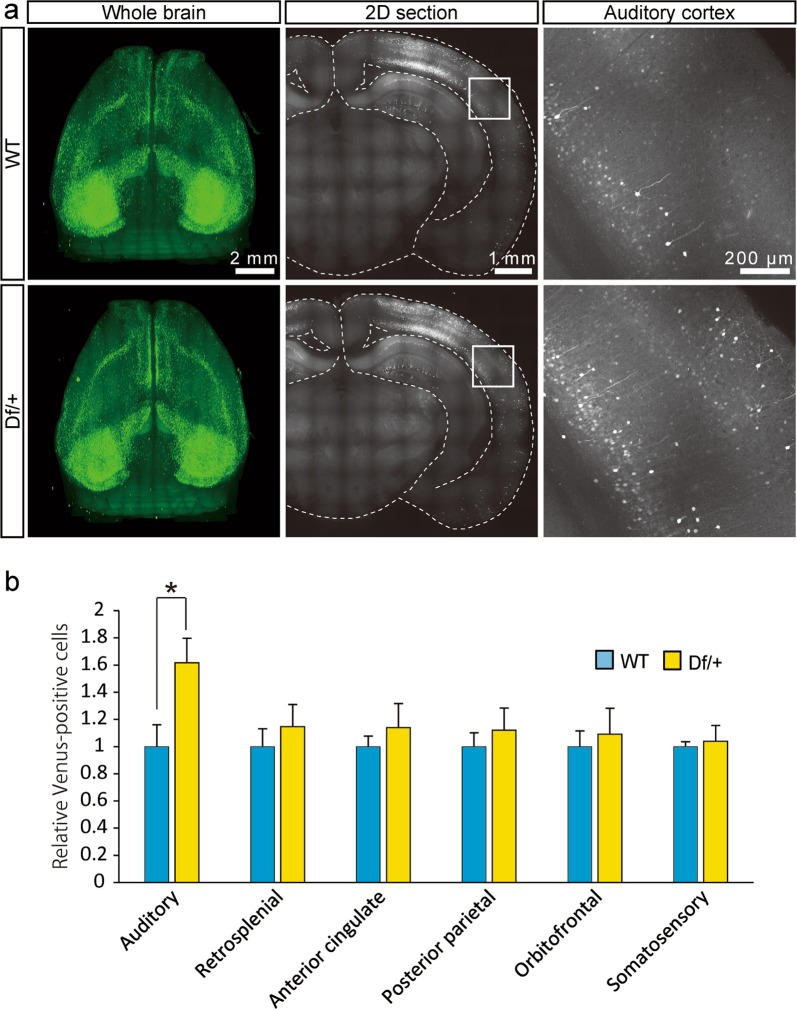
Unbiased whole-brain activation mapping after social interaction. **a** Representative whole-brain imaging of Df/+ mice and wild-type (WT) littermates carrying Arc-dVenus transgene subjected to the social interaction. Whole-brain images. Scale bar, 2 mm (left). Coronal images. Scale bar, 1 mm (center). Magnifications of the areas outlined with white boxes in the coronal images. Scale bar, 200 μm (right). **b** dVenus-positive cell counts (WT, *n* = 5; Df/+, *n* = 4). *P < 0.05, Student’s *t* test. Data are presented as the mean ± s.e.m.

We further explored the whole-brain neuronal activity patterns after specific behavioral tasks in Df/+ mice harboring the Arc-dVenus reporter gene that expresses the destabilized form of the fluorescent protein Venus (dVenus) driven by the promoter of the immediate early gene Arc [39]. Given that *Arc* was found exclusively in CaMKII-positive excitatory neurons in the cerebral cortex [44], dVenus-positive cells are suggested to be excitatory neurons. Using FAST, a high-speed serial-sectioning imaging system that we recently developed [30, 31], we unbiasedly examined dVenus expression in the whole brains of adult Df/+ mice and WT littermates and found that the activity of excitatory neurons in the auditory cortex was higher in Df/+ mice than in WT littermates after the social interaction (Fig. 4). The excitatory neural activity was also slightly increased in the retrosplenial cortex, anterior cingulate cortex, posterior parietal cortex, orbitofrontal cortex, and somatosensory cortex; however, this did not reach significant levels (Fig. 4). In other regions, such as the infralimbic cortex, piriform cortex, visual cortex, ectorhinal cortex, insular cortex, prelimbic cortex, entorhinal cortex, and motor cortex, there were no significant differences in excitatory neural activity between Df/+ mice and WT littermates (data not shown).

### Decreased PV-positive cells in the cerebral cortex in Df/+ mice

**Fig. 5:**
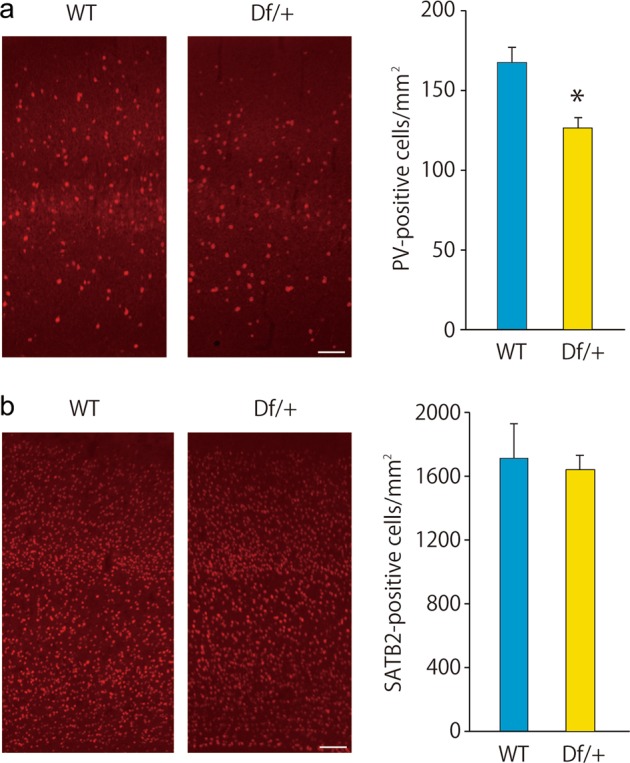
Decreased parvalbumin (PV)-positive cells in the sensory cortex in Df/+ mice. **a** PV immunostaining showing the decreased number of PV-positive cells in the adult Df/+ mice. Representative figures (left). Scale bar, 100 μm. Quantification of the number of PV-positive cells (each *n* = 3) (right). *P < 0.05, Student’s *t* test. **b** SATB2 immunostaining showing the normal number of SATB2-positive cells in adult Df/+ mice. Representative figures (left). Scale bar, 100 μm. Quantification of the number of SATB2-positive cells (each *n* = 3) (right). Data are presented as the mean ± s.e.m.

The increased excitatory neural activity in Df/+ mice may be attributable to imbalanced excitatory and inhibitory neural function. To examine this possibility, we focused on the expression levels of excitatory and inhibitory neuronal marker genes and determined that the expression level of *Pvalb* (encoding for PV) in Df/+ mice was significantly lower than that in WT littermates (Supplementary Table 1). The expression level of most of the GABAergic neuronal marker genes, except for *Slc6a13* (encoding for GAT2), was virtually similar between Df/+ mice and WT littermates (Supplementary Table 1). We also determined that the expression level of most of the excitatory neuronal marker genes was virtually similar between Df/+ mice and WT littermates (Supplementary Table 2). Although the genotype-dependent difference in the expression of *Pvalb* in the whole cortex was not significant after Bonferroni’s correction for multiple testing, these results suggest that the GABAergic system, but not the excitatory system, is impaired in Df/+ mice. To examine the decreased *Pvalb* expression in the specific brain regions, we then performed immunohistochemical analysis for PV and determined that the number of PV-positive cells was significantly decreased in the sensory cortex of Df/+ mice compared to that of WT littermates (Fig. 5a). We also performed immunohistochemical analysis for SATB2, an excitatory neural marker, and determined that the number of SATB2-positive cells was virtually similar in the sensory cortex between Df/+ mice and WT littermates (Fig. 5b). Taken together, the decreased number of PV neurons may be one of the underlying mechanisms for impaired E/I balance and hyperactivity in Df/+ mice.

## DISCUSSION

Despite substantial efforts, the molecular and cellular etiology of psychiatric disorders are largely unclear, partly because of the limited good animal models of these diseases. In this study, we generated an animal model of 3q29 deletion syndrome in which the DNA region corresponding to human 3q29 CNV was deleted. Important criteria for modeling diseases in animals are suggested to be construct validity, predictive validity and face validity [6]. Since 3q29 deletion is strongly associated with increased risk for schizophrenia as well as for intellectual disability and ASD [13–17], the Df/+ mice generated in this study satisfies construct validity. Furthermore, we determined that the mice displayed abnormalities largely relevant to psychiatric disorders and that these abnormalities were ameliorated by treatments that provide therapeutic benefit in patients with schizophrenia and ASD. These results suggest that, in addition to construct validity, Df/+ mice achieve face validity and predictive validity. Importantly, in addition to schizophrenia-related behavioral phenotypes, such as impaired PPI, ASD-related behavioral phenotypes, such as social dysfunction and increased repetitive self-grooming behavior, and impaired fear learning, Df/+ mice also showed growth defects and microcephaly, which are reminiscent of the phenotypes in patients with 3q29 deletion syndrome. Very recently, another mouse model of 3q29 deletion syndrome has been published [18]. While the genetic backgrounds between the mice and our mice are different (see below), several behavioral phenotypes, including social interaction, cognitive function and acoustic startle, and reduced body weight are reproducibly observed. In addition to these, we newly identified the cellular phenotypes of neuronal hyperactivation and the reduction of PV expression in the cortex of Df/+ mice (Figs. 3–5). Overall, the 3q29 deletion mouse model should be a key tool for unraveling the disease-causative molecular mechanism underlying the phenotypes in 3q29 deletion syndrome and, especially, for studying the developmental aspects of psychiatric disorders.

It has been shown that the genetic background of the mouse model is suggested to modify the phenotypic expression of a gene variant [6, 7, 45, 46]. Actually, while Df/+ mice (C57BL/6J background) in this study showed impaired PPI, very recently reported another mouse model of 3q29 deletion syndrome on C57BL/6N background shows normal PPI [18]. The phenotypic differences in the PPI test may be partly explained by the differences in the genetic background. It will be important to examine the phenotypes identified in this study on different genetic backgrounds and under different environments.

The important findings in this study are that the excitatory neural activity was abnormally increased in Df/+ mice (Figs. 3 and 4). Previous studies have suggested that an increased cellular E/I balance is associated with circuit hyperexcitability and psychiatric disorder-related behavioral phenotypes [28, 29, 47], although it remains controversial whether it is a causative mechanism that induced circuit hyperexcitability or a compensatory mechanism [48]. It will be interesting to further analyze cellular and circuit level electrophysiological properties in Df/+ mice so as to explore the causative mechanisms of neuronal hyperexcitability observed in Df/+ mice (Fig. 4). The limitation of this study is that, although the impaired social interaction is an important phenotype of Df/+ mice in terms of a disease model, we examined excitatory neural activity changes solely after the social interaction task. Future unbiased whole-brain imaging combined with various behaviors is important to comprehensively understand the etiology of 3q29 deletion syndrome at the brain network level.

We determined that the GABAergic system may be impaired in Df/+ mice (Fig. 5, Supplementary Table 1). GABAergic interneurons, which modulate neural circuit function through inhibition of the activity of neighboring neurons, constitute a diverse family, which are classified by their morphology, electrophysiological properties, and specific molecular markers, such as PV, somatostatin, cholecystokinin, neuropeptide Y, calretinin, and calbindin [49–51]. Among them, PV-positive interneurons play a key role in maintaining E/I balance and ultimately cognitive functions [28, 29]. Interestingly, the expression level of PV mRNA is altered in patients with schizophrenia and ASD [24, 26]. Consistent with this story, PV-knockout mice exhibit imbalanced E/I and ASD-related behavioral abnormalities [52, 53]. In Df/+ mice, the decreased PV-positive interneurons may cause imbalanced E/I, resulting in abnormal behaviors relevant to psychiatric disorders.

Approximately 22 and 24 genes are heterozygously deleted in the human 3q29 deletion and Df/+ mice, respectively (Fig. 1a). Among these gene products, Fbxo45, a ubiquitin ligase complex protein, deletion may be one of key to the developmental abnormalities because Fbxo45 is reported to be involved in neuronal development, including interneuron development and synaptic activity [54, 55]. In addition to Fbxo45, DLG1 (discs large 1), and PAK2 (Cdc42/Rac-activating kinase 2) located in this region are proposed to contribute to the phenotype of 3q29 deletion syndrome because these molecules seem to critically regulate neural function [54–57]. Deletion of other gene products, which are involved in neural development, may also be associated with the molecular etiology of 3q29 deletion syndrome. Specifically, since this study postulated that the underlying mechanism for E/I imbalance may reside primarily in PV neurons, precise gene expression analyses of 3q29 genes in PV neurons would facilitate narrowing down of the disease-causative gene(s) in 3q29 deletion syndrome. Further mechanistic studies in which each 3q29 gene is reintroduced into Df/+ mice will definitely determine which genes are causally related to the disease phenotypes and, in the long run, may lead to the identification of promising drug targets for 3q29 deletion syndrome as well as schizophrenia and ASD.

An important unresolved issue is why the phenotypes of patients with the same microdeletion are so diverse. Given that it has been hypothesized that genetic background and modifiers such as single-nucleotide polymorphisms affect the impact of the microdeletion, comprehensive genetic analysis of patients with 3q29 microdeletion syndrome will surely be an important next step to address the issue. Functional analysis of iPS cell-derived neurons from multiple patients with 3q29 microdeletion syndrome, as well as CRISPR-mediated 3q29 deletion iPS cells, would also be helpful.

Although, for generating Df/+ mice, we utilized the Cas9 nickase mutant (D10A) to greatly minimalize off-target mutagenesis [32, 33], it will be important to backcross Df/+ mice with parental C57BL/6 mice to avoid possible off-target mutations outside mouse chromosome 16.

In summary, our current findings suggest that Df/+ mice are important tools not only to unravel the causal relationship between the biological significance of genetic variants and molecular etiology of the diseases but also to provide information for the development of new effective treatment strategies for schizophrenia and ASD.

## Funding and disclosure

This work was supported in part by JSPS KAKENHI, grant numbers JP15H04645 (T.N.), JP18H02574 (T.N.), JP17K19488 (H.H.), and JP17H03989 (H.H.); the JSPS Research Fellowships for Young Scientists, a grant number JP17J00152 (K.M.); MEXT KAKENHI, grant numbers JP18H05416 (H.H.), JP19H05217 (A.K.), JP19H04909 (T.N.) and JP19H05218 (T.N.); AMED, grant numbers JP18dm0107122h0003 (H.H.), JP18dm0207061h0002 (H.H.), JP18dm0107087 (N.O.), JP18dm0207005 (N.O.), and JP18am0101084; and grants from the Takeda Science Foundation (T.N.) and Asahi Glass Foundation (T.N.). This study was also supported in part by the Center for Medical Research and Education, Graduate School of Medicine, Osaka University.

## Supplementary information

Supplemental Material
