# Supplementary material for: Psychiatric-disorder-related behavioral phenotypes and cortical hyperactivity in a mouse model of 3q29 deletion syndrome
Source: Neuropsychopharmacology. 2019 Jun 19;44(12):2125–35. doi: 10.1038/s41386-019-0441-5 (PMC6887869; doi:10.1038/s41386-019-0441-5)
Supplement: Supplementary file 1 — Supplemental Material [file 41386_2019_441_MOESM1_ESM.pdf]

Supplementary Table 1. Expression of GABAergic neuronal markers in the mouse cortex.

Gene	WT (mean FPKM)	Df/+ (mean FPKM)	*P* value
marker			
Pvalb	61.19 ± 1.447	51.99 ± 0.816	***0.017***
Cck	296.2 ± 10.33	283.0 ± 8.017	0.718
Sst	321.5 ± 11.97	319.8 ± 14.19	0.400
Vip	15.89 ± 0.289	15.21 ± 0.661	0.557
transporter			
Slc6a1 (GAT1)	106.8 ± 3.325	104.0 ± 2.059	0.580
Slc6a13 (GAT2)	6.534 ± 0.344	5.219 ± 0.181	***0.021***
Slc6a11 (GAT3)	76.22 ± 4.705	71.73 ± 1.024	0.734
Slc32a1 (VGAT)	54.50 ± 0.963	52.89 ± 2.777	0.959
decarboxylase			
Gad2 (GAD65)	42.16 ± 0.335	41.73 ± 0.471	0.484
Gad1 (GAD67)	126.4 ± 2.821	121.1 ± 2.244	0.971
receptor			
Gabra1 ($GABA_A$ α1)	81.84 ± 0.781	77.84 ± 1.421	0.745
Gabrb1 ($GABA_A$ β1)	10.99 ± 1.424	10.87 ± 0.356	0.914
Gabrg1 ($GABA_A$ γ1)	5.676 ± 0.344	5.453 ± 0.174	0.552
Gabbr1 ($GABA_B$ 1)	142.4 ± 1.935	140.4 ± 1.432	0.068
Gabbr2 ($GABA_B$ 2)	54.28 ± 1.297	53.96 ± 0.659	0.295

Mean FPKM values are calculated from 3 mice. Data are presented as the mean ± s.e.m.

Note that *P* value is not corrected.

Supplementary Table 2. Expression of excitatory neuronal markers in the mouse cortex.

Gene	WT (mean FPKM)	Df/+ (mean FPKM)	*P* value
transporter			
Slc17a7 (VGLUT1)	342.4 ± 2.614	346.3 ± 9.265	0.067
Slc17a6 (VGLUT2)	18.46 ± 1.111	17.76 ± 0.380	0.820
Slc17a8 (VGLUT3)	0.981 ± 0.061	0.872 ± 0.040	0.189
glutamine synthetase			
Glul	475.6 ± 25.85	430.6 ± 9.038	0.375
receptor			
Gria1 (GluA1)	75.03 ± 0.950	76.99 ± 0.355	0.102
Gria2 (GluA2)	91.46 ± 6.512	97.87 ± 4.338	0.325
Grin1 (GluN1)	120.7 ± 1.756	126.1 ± 2.951	***0.006***
Grin2a (GluN2A)	8.728 ± 1.349	10.81 ± 0.054	0.271
Grin2b (GluN2B)	8.021 ± 1.996	10.63 ± 0.922	0.348

Mean FPKM values are calculated from 3 mice. Data are presented as the mean ± s.e.m. Note that *P* value is not corrected.

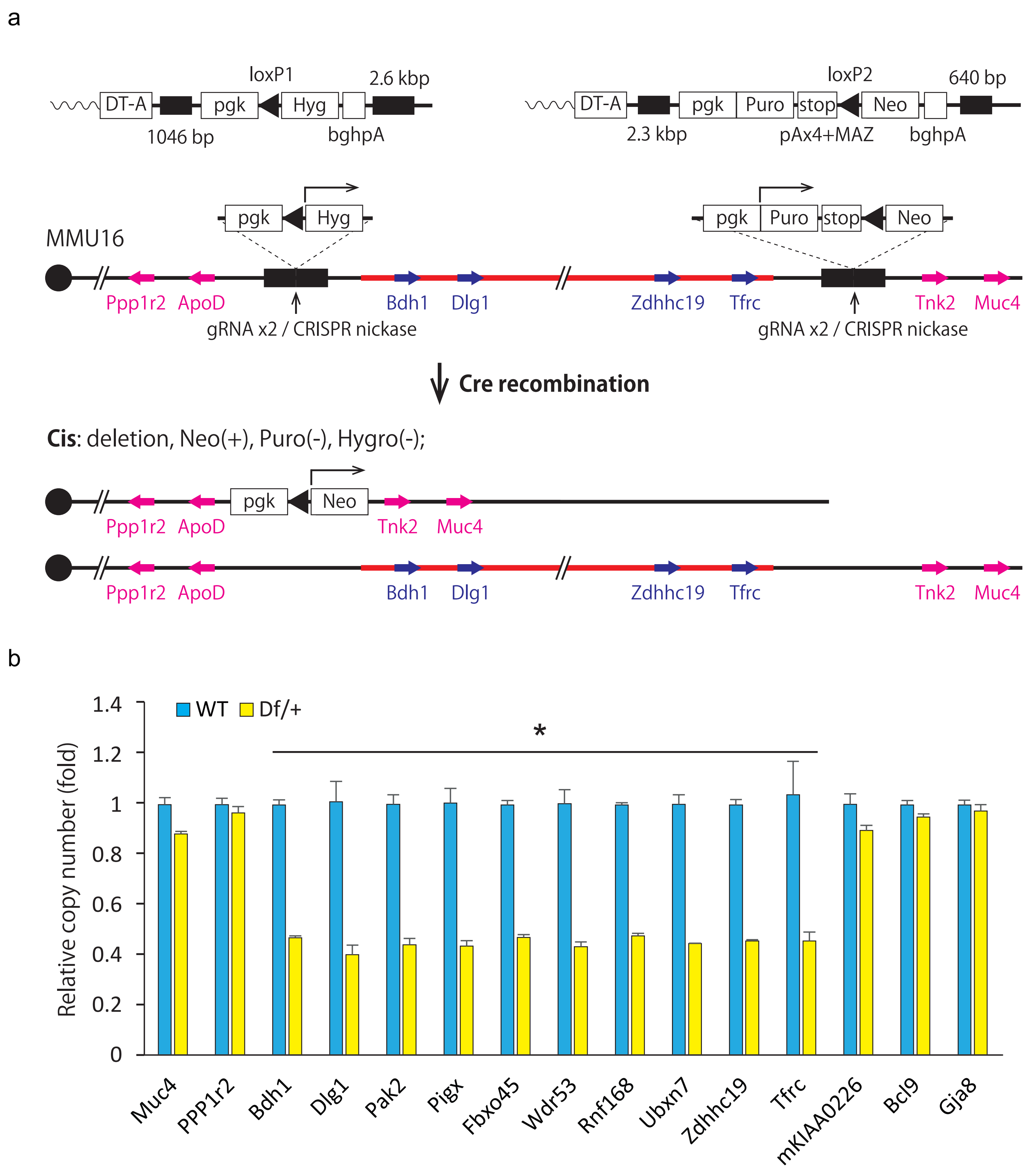

Supplementary Figure 1. Baba et al.

Supplementary Fig. 1 Generation and characterization of Df/+ mice. **a** Schematic of the strategy used to generate Df/+ mice. For details, see Materials and Methods. **b** Decreased genomic copy number of the *Bdh1-Tfrc* region in Df/+ mice. Copy number analysis of the *Bdh1-Tfrc* region was performed by genomic qPCR analysis.
